# Emergency Medicine Resident Perceptions About the Need for Increased Training in Communicating Diagnostic Uncertainty

**DOI:** 10.7759/cureus.2088

**Published:** 2018-01-19

**Authors:** Kristin L Rising, Dimitrios Papanagnou, Danielle McCarthy, Alexzandra Gentsch, Rhea Powell

**Affiliations:** 1 Department of Emergency Medicine, Thomas Jefferson University; 2 Department of Emergency Medicine, Northwestern University Feinberg School of Medicine; 3 Department of Medicine, Thomas Jefferson University

**Keywords:** diagnostic uncertainty, graduate education, training, residency, communication, emergency medicine

## Abstract

Introduction

Diagnostic uncertainty is common in healthcare encounters. Effective communication is important to help patients and providers navigate diagnostic uncertainty, especially at transitions of care. This study sought to assess the experience and training of emergency medicine (EM) residents with communication of diagnostic uncertainty.

Methods

This was a survey study of a national sample of EM residents. The survey questions elicited quantitative and qualitative responses about experiences with and educational preparation for communication with patients in the setting of diagnostic uncertainty.

Results

A sample of 263 emergency medicine residents who had trained at over 87 medical schools and 37 residency programs responded to the survey. Nearly half of participants noted they frequently encountered challenges with these conversations;  63% reported having been “somewhat” or less trained to have these conversations during residency, and 51% expressed a strong desire for more training in how to approach these discussions. Survey respondents reported that prior educational experiences in the communication of diagnostic uncertainty were largely informal and that many residents experience frustration in clinical encounters due to inability to meet patients’ expectations of reaching a diagnosis at the time of discharge.

Conclusion

This study found that emergency medicine residents frequently struggle in communicating with patients when there is diagnostic uncertainty upon emergency department discharge and perceived the need for training in how to communicate in these situations. The development of targeted educational strategies for improving communication in the setting of diagnostic uncertainty is consistent with emergency medicine core competencies and may improve patient and provider satisfaction with these clinical encounters.

## Introduction

Uncertainty is inherent to the diagnostic process. Establishment of a diagnosis occurs over time, commonly beginning when a patient notes symptoms and presents for care, and proceeding as an iterative cycle of information gathering, interpretation, and synthesis to develop a set of working diagnoses and ultimately a final diagnosis [1]. As clinicians and patients work together to establish a diagnosis and develop a management plan, they face various degrees of uncertainty across a number of points in time. Clear and effective communication between patients and providers about diagnostic uncertainty is necessary to empower patients to safely manage their health throughout this process.

Diagnostic uncertainty impacts most healthcare settings and is especially common in the emergency department (ED). One study estimated that at least 37% of patients discharged from the ED are discharged with a “symptom-based diagnosis” rather than a pathologic diagnosis [2]. For example, a patient may present to the ED with chest pain, have an evaluation that rules out any immediately threatening conditions, and be discharged for further outpatient workup without identifying the cause of their chest pain. Clinicians’ and patients’ responses to uncertainty differ and can impact clinician practice patterns and patient experience. Patients facing ongoing uncertainty about symptoms after an ED discharge report fear and frustration [3-4]. Clinicians experience varying levels of discomfort related to an uncertainty that is influenced by their demographics and is associated with differences in practice patterns [5]. One study found an association between reduced tolerance of uncertainty and increased patient charges [6]; other work has found that increased need for cognitive closure among obstetrician and gynecologists was associated with asking well-visit screening questions less frequently and fewer referrals to specialists [7]. Clinicians have been shown to modify the degree of uncertainty communicated based on their perception of a patient’s aversion to ambiguity [8]. In the outpatient setting, although the diagnostic process yields a specific diagnosis for many patients, others experience ongoing symptoms despite normal test results [9-11]. Patients in this setting may not be reassured by a normal evaluation and can experience heightened levels of anxiety from unexplained symptoms.

Despite the presence of uncertainty across healthcare disciplines and the discomfort that uncertainty can cause for both patients and clinicians, there is no clear guidance or best practice for educating healthcare providers on how to approach communicating diagnostic uncertainty with patients. The overall objective of this work was to demonstrate the current gap in educational training with regards to communicating diagnostic uncertainty. To do so, we engaged emergency medicine (EM) residents to study their reported perceptions of the training they have received regarding communication of diagnostic uncertainty, the challenges they have faced communicating uncertainty with patients, and their interest in receiving future formalized training in the communication of diagnostic uncertainty.

## Materials and methods

Study design

This was a survey of a national sample of emergency medicine residents throughout the United States in the 2016 academic year, designed to elicit both quantitative and qualitative responses. The survey included basic demographic questions followed by four questions linked to five-point Likert scale response options and an optional open-ended comment box after each question. The first question asked about clinical experiences communicating diagnostic uncertainty, the next two asked about training received in medical school or residency on the communication of diagnostic uncertainty, and the final question asked about the desire for formal training on the communication of diagnostic uncertainty. Survey questions are listed in the Appendix. 

Participants

Potential participants were all current EM resident trainees (post-graduate years (PGY) I-IV) from accredited United States (US) EM residency programs with no exclusion criteria. EM residency programs vary in length (i.e., three years versus four years) based on the sponsoring hospital and/or academic institution. To capture findings across both program types, residents in both three-year and four-year EM programs were invited to participate. An invitation to participate and a link to the survey was distributed through the Council of Emergency Medicine Residency Directors (CORD) listserv, which includes residency program leadership (i.e., program directors, assistant/associate program directors, and student clerkship directors) from US training programs in EM that are accredited by the Accreditation Council for Graduate Medical Education (ACGME) or the American Osteopathic Association (AOA). Recipients of this correspondence were asked to forward the survey link to their respective trainees. While it was impossible to determine how many program leaders sent the survey to their respective trainees, weekly reminders were sent to the CORD listserv during the six weeks that the survey was active. 

Survey development

The survey was developed by the study investigators with the intended goals of a) assessing the training EM residents have had in communicating diagnostic uncertainty to their patients and b) assessing EM residents' self-perceived need for training to effectively communicate diagnostic uncertainty to their patients. Survey items were adapted from previous works that examined physicians' reactions to uncertainty [5-6, 8]. The survey was piloted anonymously with a group of EM residents at Thomas Jefferson University Hospital in Philadelphia, PA, which offers a three-year EM residency program accredited by the ACGME.

Survey questions, which were kept short and concise, underwent three rounds of review by the authors to ensure clarity of language and adherence to the goals of the study. In addition, instructions were included in the survey for participants that clearly stated the intentions of the research investigation.

Data collection

The survey was developed with Qualtrix® software (Qualtrix, Provo, UT), and distributed via email through the CORD listserv as described above. Survey responses were anonymous unless respondents opted to provide contact information in order to be contacted about future prospective work on this topic. The survey link was open for six weeks from the initial invitation with automated weekly reminder emails. The survey was optional, with no personal health or other identifiable information collected, and participation in the survey was considered participant assent. The study was approved by the Institutional Review Board (IRB) of the Sidney Kimmel Medical College of Thomas Jefferson University in Philadelphia, Pennsylvania (approval # 16E.484). 

Data analysis

We provide simple descriptive statistics for all survey responses. The study was not designed to capture how many individuals received and completed the survey; consequently, a response rate is not reported. Three members of the research team analyzed all qualitative data extracted from the optional comment-based survey items with a content analysis approach [12]. All coding and analysis were performed by group consensus.

## Results

Participant characteristics

A total of 263 participants provided at least partial survey responses; there were 240 participants in the survey who provided demographic information (Table 1). Mean participant age was 30 (range: 25-44), with 41% females. Almost two-thirds of participants were in one of the first two years of residency training, and there was a close distribution in respondents from three-year and four-year residency programs (54% and 46%, respectively). Participants represented at least 87 medical schools and 37 residency programs (answers to these questions were optional).

**Table 1:** Participant Demographics (n = 240) n: number

Participant Characteristic	n (%)
Age – mean (range)	30 (25-44)
Female	97 (41%)
Residency Year 1 2 3 4 >4	83 (35%); 70 (29%); 51 (21%); 29 (12%); 7 (3%), respectively
Type of Emergency Medicine Program: 3 year 4 year	130 (54%); 109 (46%), respectively

Prior challenges communicating diagnostic uncertainty

Participants were asked whether they had encountered any challenges when discharging patients with diagnostic uncertainty. Fifty-three percent of the sample (138/263) reported "sometimes" encountering challenges in discharging patients with diagnostic uncertainty in contrast to 43% (114/263) who reported encountering these challenges "often" or "always" and 1% (3/263) who reported "never" encountering these challenges.

There were 101 free text responses to this first set of questions in which resident physicians provided comments describing their clinical experiences with challenges in communicating diagnostic uncertainty. Responses were categorized into three main thematic categories: patient responses to uncertainty, provider (resident) responses to uncertainty, and communication challenges.

Participant comments about patient responses to uncertainty focused on patient expectations and trust in the medical system. Participants described patients as often arriving to the ED with an expectation of receiving answers and leaving 'angry', 'frustrated', 'confused', 'upset', 'unsatisfied', 'unhappy', 'disappointed', and 'concerned' when they did not receive a definitive diagnosis and/or answers to their questions. Participants noted experiences in which patients did not want to leave the ED until a definitive diagnosis was provided. As one participant explained, “Patients want definitive answers and inconclusive is the opposite.” They also discussed the perceived need for reassurance of patients in the ED; for example: “As uncomfortable as we are with uncertainty, our patients are often even more uncomfortable. They WANT reassurance.”

Multiple participants reported perceiving mistrust in the medical system among patients who were discharged without a definitive diagnosis: “Patients believe they have not received thorough care if they don't have a named diagnosis.” Residents’ comments described patient confusion when the ED workup concluded without a definitive diagnosis, and patient discontent with outpatient referral for additional evaluation:

“Some patients get upset when I have to communicate to them that the remainder of their medical problems need to be worked up on an outpatient basis. I've never asked why, but I think they feel like I am punting them to another provider.”

Resident participants noted their own emotional responses to uncertainty were sometimes related to logistics potentially impacting patient follow-up. Residents described concerns about whether patients would be able to successfully receive needed follow-up care. One participant highlighted the challenges associated with providing clear follow-up instructions for patients who did not receive a specific diagnosis. Many of the participants acknowledged their own discomfort “wondering if I am missing something, thinking about the patient feeling frustrated that I don’t know the diagnosis, wondering if the patient is going to ‘fly’ at home.” Several participants noted feeling a sense of failure as a provider when they were not able to give their patients a diagnosis. Residents also described their logistical concerns about whether patients would be able to successfully receive follow-up care, with one participant highlighting the challenges associated in providing patients with clear follow-up instructions for those who did not receive a diagnosis.

Finally, participants reported on specific communication issues they had experienced with conversations involving diagnostic uncertainty. They discussed challenges with “convincing (patients) that the good news is that the results are not positive”, noting that patients without a clear diagnosis often did not feel reassured at their time of discharge, even if a serious disease was ruled out. Participants also described communication problems encountered when they explained to patients that an outpatient workup (versus an inpatient admission) was the appropriate next step. Participants noted difficulty in providing anticipatory guidance for patients with diagnostic uncertainty, as they could not identify the etiology of patients’ symptoms and therefore could not predict the course of illness. Finally, participants described communication challenges when discussing the limitations of the ED workup with their patients and why it may sometimes be appropriate for patients to leave the ED without specific answers so long as their respective work-ups excluded life-threatening pathologies.

Prior medical school and residency training

**Figure 1:**
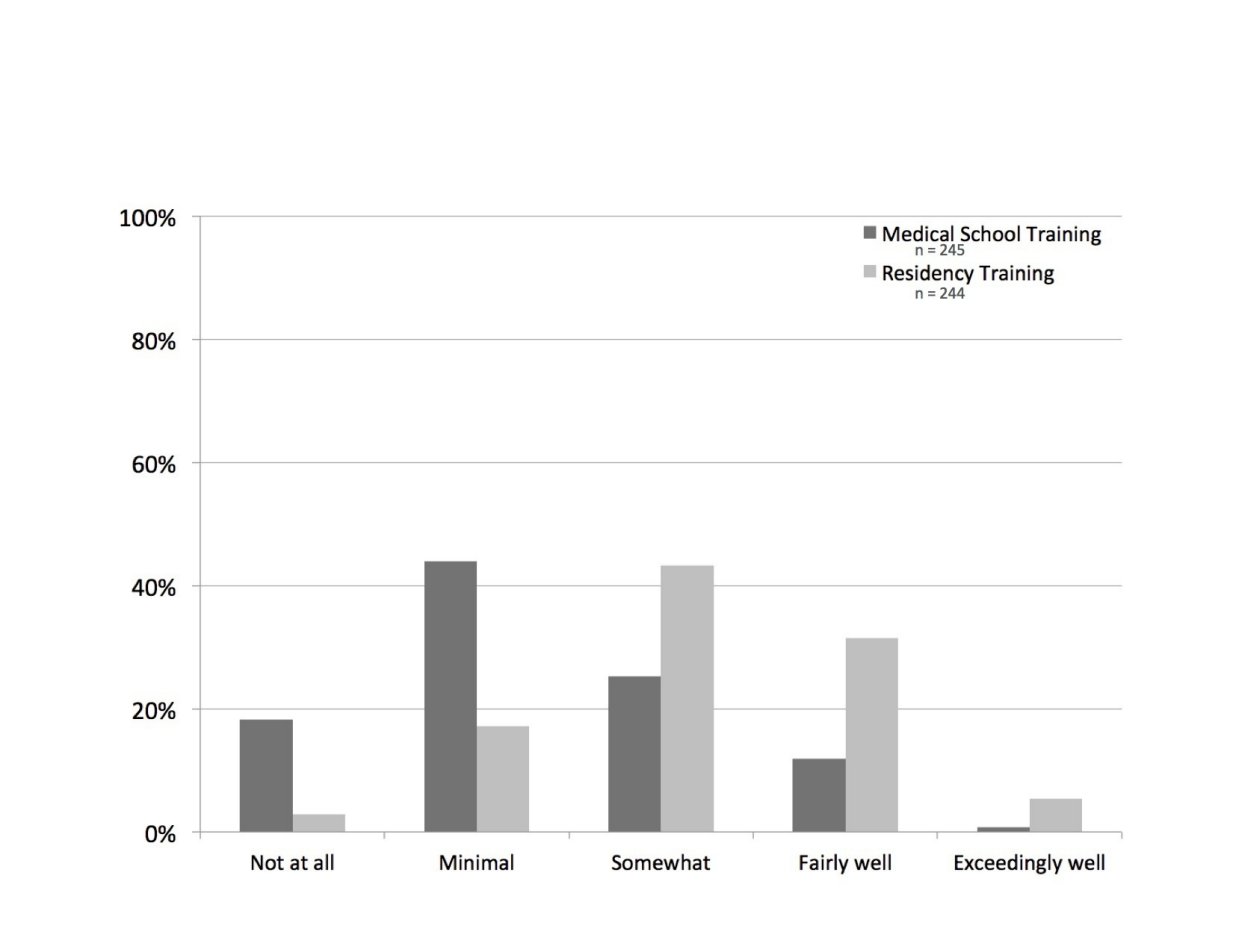
Preparation and Training for Conversation About Diagnostic Uncertainty n: number

Participants were then asked how well medical school training and residency training in EM had prepared them for having conversations regarding diagnostic uncertainty (Figure 1). Participants who reported receiving at least some training were also asked to note whether training was formal (i.e., as part of a course, curriculum, and/or lecture), informal, or both. The majority (62%, 157/253) reported medical school training had prepared them “not at all” or “minimally”, compared to 13% (32/253) who felt they had been prepared “fairly well” or “exceedingly well”. Of those receiving at least some training in medical school, two-thirds (70%, 118/169) responded that this training was received informally. With regards to residency training, over one-third (37%, 90/245) reported being trained “fairly well” or “exceedingly well”, with the largest portion of participants being “somewhat” trained (43%, 106/245). Similarly, the majority of training (85%, 187/220) received in residency was informal.

**Figure 2:**
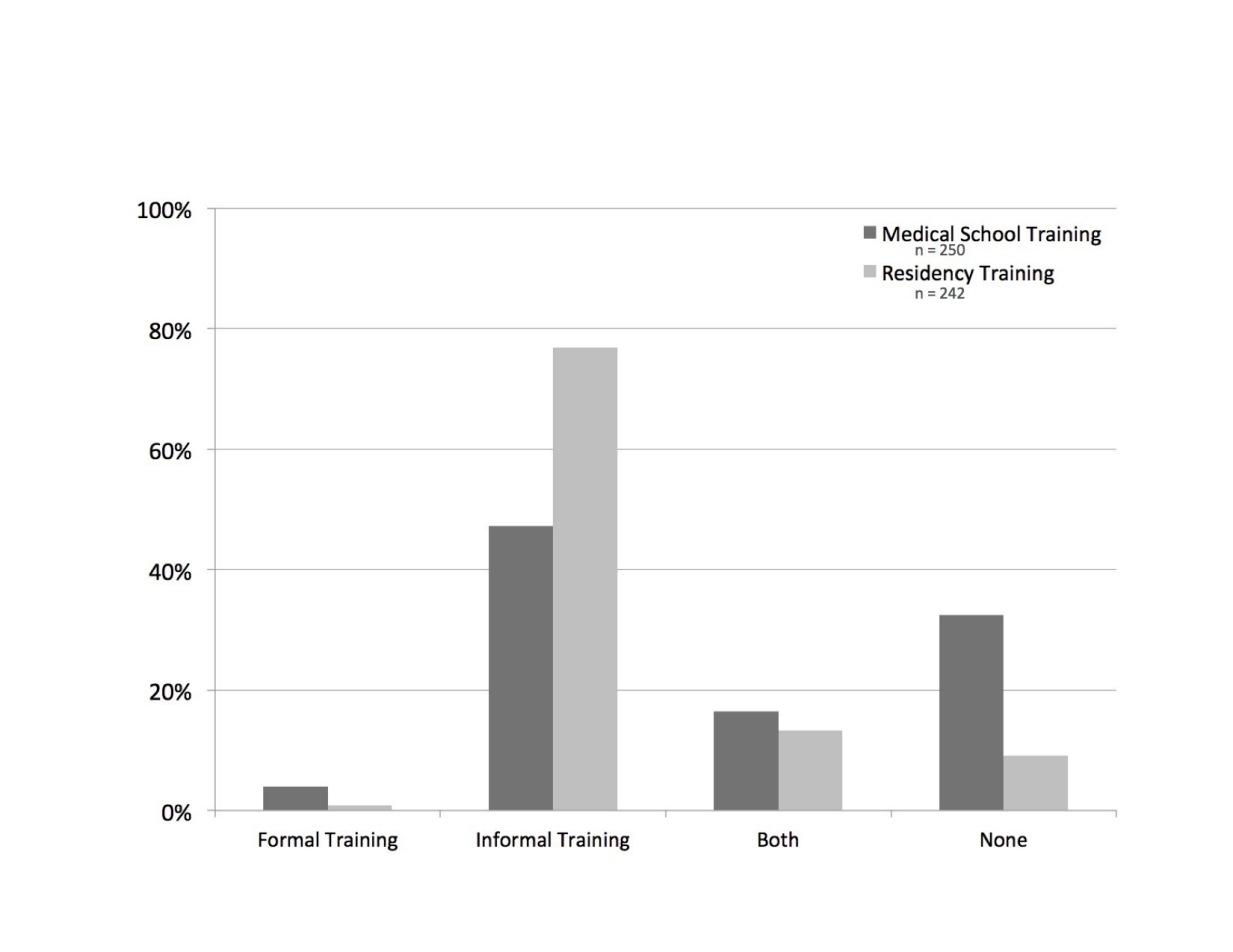
Types of Training for Conversations About Diagnostic Uncertainty n: number

Forty-four comments were provided about the types of training participants received, either in medical school or residency. The majority of comments reinforced that any training received around diagnostic uncertainty, both in medical school and residency, was informally received through direct patient care (Figure 2). Participants reported that helpful patient care experiences were those in which they observed faculty and/or residents modeling these conversations with their patients. This was highlighted in one respondent’s comment: “Most of the informal training depends on my attending's level of comfort with diagnostic uncertainty. Some attendings are comfortable with it, and some are not.” Several residents also noted that they learned how to navigate these conversations through their own experiences with direct patient care; as stated by one resident, “I am learning how to do it in residency out of necessity.”

A few participants identified formal classroom training in either medical school or residency that was germane to discussing diagnostic uncertainty. In medical school, this training was in the format of “doctoring” courses, which focused on basic clinical skills (i.e., general doctor-patient communication; presenting uncomfortable topics to patients; communicating inconclusive results). In residency, participants reported receiving this training in a small group or lecture format. Participants also reported having received relevant formal training in medical school through simulation-based or standardized-patient encounters. Participants described simulation cases in which they had to provide “awkward” or uncomfortable information to patients, break bad news, or address difficult patients; in this subset of respondents, these experiences were helpful training opportunities for how to comfortably communicate diagnostic uncertainty.

Perceived need for future training

Finally, participants were asked about their perceived need for additional training to prepare them to engage in a dialogue with patients with whom there was diagnostic uncertainty. More than half of the sample (51%, 125/244) expressed a strong desire for additional formal training, with the remainder of the sample feeling neutral on the topic (24%, 58/244) or feeling that additional training is not required (25%, 61/244).

There were 20 free-text responses to this question. These responses expressed general thoughts on why training was needed, suggestions for possible training, and opinions on potential barriers to effective training. Additional training in this area was deemed necessary due to the frequency of clinical encounters with diagnostic uncertainty, observed conversations where there was clear miscommunication between providers and patients regarding the outcomes of a visit, and the acknowledgment that providers can always learn to be better communicators. Participants suggested that, overall, training should focus on general communication skills, as well as empowering trainees to acknowledge: "I don't know, and there's no test that will give me 100% certainty." A few residents suggested that illustrative cases would be helpful, potentially provided through short videos. Most felt that such training should be short and concise, and effectively equip them with the syntax (i.e., a few sentences) and/or skills to apply during future patient encounters. The primary concern regarding potential barriers to training was that “it's kind of a nebulous subject that is hard to pin down and concretely discuss,” leading a few participants to express the opinion that structured training programs would not be very helpful.

## Discussion

We surveyed a sample of EM residents across accredited residency training programs regarding their experience with the communication of diagnostic uncertainty to patients at the time of ED discharge. Close to half of participants noted “often” or “always” encountering challenges during these conversations, close to two-thirds (63%) reported being “somewhat” or less trained by their residency to appropriately conduct these conversations, and over half (51%) expressed a strong desire for additional training on how to approach these discussions.

Previous research has demonstrated that over one-third of patients leave the ED with diagnostic uncertainty [2] and uncertainty is distressing to patients [3-4, 13-14]. The comments provided in response to our survey corroborate these findings and further highlight that communicating diagnostic uncertainty can be challenging and distressing to providers. Adequate communication is a primary element for high-quality, safe ED discharges. Specifically, communication of the discharge diagnosis, prognosis, treatment plan, and anticipated disease course are considered key elements to a high-quality ED discharge [15]. Prior work on communication during care transitions has focused on improving the content, delivery, and comprehension of discharge instructions [16-17], including the provision of diagnosis-specific discharge instructions where possible [18-19]. For patients discharged from the ED with diagnostic uncertainty, no standardized instructions exist. Our work suggests that guidance and training are needed for communicating discharge plans in the setting of diagnostic uncertainty.

Effective patient communication is a core competency for EM residency training [15-21]. Though patient-centered communication skills and the ability to establish a comprehensive and appropriate discharge plan are core competencies for medical residents [21], there are no widely implemented approaches for teaching resident physicians how to communicate most effectively with patients in encounters with diagnostic uncertainty (Table 2). Our work suggests that EM residents want and need guidance on how to navigate these clinical encounters. Our survey findings confirm that enhanced healthcare professional education and training is needed to build competency in the communication of diagnostic uncertainty and the diagnostic process [1, 22].

**Table 2:** Emergency Medicine Competencies Related to Topic of Diagnostic Uncertainty ACGME: Accreditation Council for Graduate Medical Education

Competency, brief description of skill, and ACGME milestone targets for skill levels of competency						
Patient Care #7: Disposition Competency: Establishes and implements a comprehensive disposition plan that uses appropriate consultation, patient education regarding diagnosis, treatment plan, medications, and specific disposition instructions.						
Level 1 - Describes basic resources available for care	Level 2 - Formulates a specific follow-up plan for common emergency department complaints	Level 3 - Formulates and provides patient education regarding diagnosis, treatment plan, and medication review - Involves appropriate resources in a timely manner		Level 4 - Formulates discharge instructions, including future diagnostic/therapeutic interventions for emergency department patients - Engages patient to effectively implement a discharge plan		Level 5 - Works within the institution to develop systems that enhance safe disposition
Interpersonal and Communication Skills #1: Patient-Centered Communication Competency: Demonstrates interpersonal and communication skills that result in the effective exchange of information and collaboration with patients and their families.						
Level 1 - Establishes rapport - demonstrate empathy - Listens effectively	Level 2 - Elicits patients’ reasons for seeking health care and expectations from the emergency department visit - Negotiates and manages simple conflicts		Level 3 - Manages the expectations of emergency department patients and uses communication methods that minimize the stress, conflict, and misunderstanding - Effectively communicates with vulnerable populations		Level 4 - Uses flexible communication strategies and adjusts based on the clinical situation to resolve specific emergency department challenges	Level 5 - Teaches communication and conflict management skills - Participates in review and counsel of colleagues with communication deficiencies

Providers in many specialties would likely benefit from such training; however, our findings also suggest that there are potentially unique challenges in the ED environment. Mismatched expectations between patients and providers about the capabilities of the ED are commonly noted in this setting. The Disconfirmation Paradigm (developed within the non-medical customer satisfaction literature) posits that when service perceptions fall short of expectations, dissatisfaction ensues [23]. Previous studies within the EM patient satisfaction literature have applied the disconfirmation paradigm to patient expectations of wait times and throughput times finding that patients are less satisfied when their expectations are not met [24-25]. It is likely that the mismatch of perceptions and expectations on the topic of diagnostic uncertainty also influences patients’ satisfaction with their ED experience through negative disconfirmation.

Limitations

There are several limitations to this study. Participants were solicited through a residency director email listserv, and thus it is likely that entire residency programs did not receive the link for study participation. We are unable to determine which residency directors did forward the link and whether there are systematic differences in the programs who participated and who did not participate. Consequently, a response rate could not be reported.

In addition, it is possible there was self-selection bias by residents who were more invested in this topic, thus potentially skewing results toward a stronger sense of lack of training and the need for more training than exists across the population as a whole. Similarly, residents may have been more likely to provide illustrative examples of discharge conversations that went poorly than of those that were successful or well-received by patients. It also was not possible to cluster results by residency program, geographic region, or hospital setting; this would be beneficial in subsequent studies to further clarify any relationships that may exist across various practice settings (i.e., urban vs. rural hospitals, academic vs. community programs). 

## Conclusions

These findings demonstrate that EM residents frequently struggle with discharging patients for whom there is diagnostic uncertainty and that the majority of residents perceive a need for additional training in how to communicate during these difficult situations. Effective patient communication is paramount to addressing diagnostic uncertainty. The development of training opportunities on effective communication regarding diagnostic uncertainty is congruent with core competencies in EM resident education, and improvement in conducting these discharge conversations will lead to safer transitions of care and higher patient satisfaction. Future work in this area is needed to develop and test effective educational strategies that will assist clinical providers with navigating these difficult conversations with their patients.

## Appendices

Communicating Uncertainty: Survey Questions

Q1: Have you encountered any challenges when discharging patients with diagnostic uncertainty?

o Never

o Rarely

o Sometimes

o Often

o All the time

Q2: If so, what have been some of these challenges (optional)?

________________________________________________________________

Q3: How well do you feel your medical school training prepared you for having conversations with patients about diagnostic uncertainty?

o Not at all

o Minimally

o Somewhat

o Fairly well

o Exceedingly well

Q4: If you received such training in medical school, was this training:

o Formal training

o Informal training

o Both formal and informal training

o Other (please describe) ________________________________________________

o I did not receive this training in medical school.

Q5: Please feel free to provide any comments here regarding your training in medical school on having conversations with patients about diagnostic uncertainty (optional). 

________________________________________________________________

Q6: How well do you feel your residency training has prepared you so far for having conversations with patients about diagnostic uncertainty?

o Not at all

o Minimally

o Somewhat

o Fairly well

o Exceedingly well

Q7: If you received such training in residency, was this training:

o Formal training

o Informal training

o Both formal and informal training

o Other (please describe) ________________________________________________

o I have not received this training in residency.

Q8: Please feel free to provide any comments here regarding your training in residency on having conversations with patients about diagnostic uncertainty (optional).

________________________________________________________________

Q9: How much do you agree with the following statement: “I need more formal training to prepare me to speak with patients about diagnostic uncertainty."

o Strongly disagree

o Moderately disagree

o Neutral

o Moderately agree

o Strongly agree

Q10: Please feel free to provide any comments you may have regarding diagnostic uncertainty here (optional).

________________________________________________________________

Please note: The questions that follow ask for demographic and program-specific information:

Q11: What type of emergency medicine residency training program are you enrolled in?

o 3-Year Program

o 4-Year Program

Q12: What is your current year of residency training?

o 1

o 2

o 3

o 4

o More than 4 (please specify) ____________________________________________

Q13: What is your age?  _______________________________________________________

Q14: Please select your gender:

o Female

o Male

o Other (please specify) ________________________________________________

o Prefer not to answer

Q15: What is the name of the medical school you attended (optional)?

________________________________________________________________

Q16: What is the name of your residency program (optional)?

________________________________________________________________

Q17: What is your name (optional)?

________________________________________________________________

Q18: Are you interested in being part of a workgroup to develop a medical education curriculum on best practices for addressing diagnostic uncertainty with patients?

o No

o Yes (Please provide your email address here to be contacted) ____________________
